# A combination of TLR-4 agonist and saponin adjuvants increases antibody diversity and protective efficacy of a recombinant West Nile Virus antigen

**DOI:** 10.1038/s41541-018-0077-1

**Published:** 2018-09-26

**Authors:** Neal Van Hoeven, Steven Wiley, Emily Gage, Andrew Fiore-Gartland, Brian Granger, Sean Gray, Christopher Fox, David E. Clements, D. Elliot Parks, Scott Winram, Dan T. Stinchcomb, Steven G. Reed, Rhea N. Coler

**Affiliations:** 1 Infectious Disease Research Institute, 1616 Eastlake Ave E., Suite 400, Seattle, WA 98102 USA; 2 Pathobiology Program, Department of Global Health, University of Washington, Seattle, WA 98195 USA; 3 Imdaptive Inc., 3010 Northwest 56th Street, Seattle, WA 98107 USA; 4 Vaccine and Infectious Disease Division, Fred Hutchinson Cancer Research Center, 1100 Fairview Ave, Seattle, WA 98109 USA; 5 PAI Life Sciences Incorporated, 1616 Eastlake Avenue, Suite 250, Seattle, WA 98102 USA; 6 Hawaii Biotech Inc., 99-193 Aiea Heights Drive, Aiea, HI 96701 USA; 7 Leidos Inc., 11951 Freedom Drive, Reston, VA 20190 USA

## Abstract

Members of the *Flaviviridae* family are the leading causes of mosquito-borne viral disease worldwide. While dengue virus is the most prevalent, the recent Zika virus outbreak in the Americas triggered a WHO public health emergency, and yellow fever and West Nile viruses (WNV) continue to cause regional epidemics. Given the sporadic nature of flaviviral epidemics both temporally and geographically, there is an urgent need for vaccines that can rapidly provide effective immunity. Protection from flaviviral infection is correlated with antibodies to the viral envelope (E) protein, which encodes receptor binding and fusion functions. TLR agonist adjuvants represent a promising tool to enhance the protective capacity of flavivirus vaccines through dose and dosage reduction and broadening of antiviral antibody responses. This study investigates the ability to improve the immunogenicity and protective capacity of a promising clinical-stage WNV recombinant E-protein vaccine (WN-80E) using a novel combination adjuvant, which contains a potent TLR-4 agonist and the saponin QS21 in a liposomal formulation (SLA-LSQ). Here, we show that, in combination with WN-80E, optimized SLA-LSQ is capable of inducing long-lasting immune responses in preclinical models that provide sterilizing protection from WNV challenge, reducing viral titers following WNV challenge to undetectable levels in Syrian hamsters. We have investigated potential mechanisms of action by examining the antibody repertoire generated post-immunization. SLA-LSQ induced a more diverse antibody response to WNV recombinant E-protein antigen than less protective adjuvants. Collectively, these studies identify an adjuvant formulation that enhances the protective capacity of recombinant flavivirus vaccines.

## West Nile Virus: adjuvant combinations boost vaccine efficacy

There is currently no approved human vaccine for West Nile Virus (WNV); however, it is known that protective immune responses are generally directed to the viral E protein. Neal Van Hoeven and colleagues at the Infectious Disease Research Institute in Seattle use recombinant WNV E-protein antigen adjuvanted with different combinations of a synthetic Toll-like receptor 4 agonist (SLA) and the saponin QS21 to determine optimal vaccination strategies in preclinical mouse and hamster models. SLA plus QS21 synergize in the production of neutralizing antibodies and when used together generate higher antibody diversity—a property that directly correlates with their protective capacity in vivo. Uniquely, the combination of QS21 plus SLA was also able to elicit robust T helper 1 responses. These findings highlight a promising adjuvant combination that might form the basis of an effective human WNV vaccine.

## Introduction

Members of the *Flaviviridae* family of arboviruses cause significant morbidity and mortality throughout the world. Dengue virus (DENV) causes as estimated 360 million cases/year^1^ while yellow fever virus (YFV) continues to cause local epidemics that strain the stockpiles of an effective vaccine. Other members of the family including West Nile Virus (WNV) and Zika virus (ZIKV) have emerged to cause widespread outbreaks in naïve populations, with significant morbidity and mortality due to the neurotropism of these viruses. Licensed vaccines for flaviviruses include live attenuated viruses (YF17D for yellow fever, SA14.14.2 for Japanese encephalitis virus (JEV)), recombinant chimeric viruses (DengVaxia, for DENV, ChimeriVax-JE for JEV), and inactivated whole virus vaccines (e.g. Ixiaro for JEV, FSME-IMMUN and Encepur for tick-borne encephalitis virus). While effective, these approaches have long development cycles and have manufacturing challenges which can restrict available vaccine supply.^2^ In addition to these traditional approaches, recombinant subunit vaccines targeting the envelope (E) protein have been tested in preclinical studies and in Phase 1 clinical trials. We have previously described a novel WNV vaccine formulation containing a recombinant E-protein combined with a TLR agonist adjuvant.^3^

While the global burden of WNV disease is difficult to estimate due to lack of reporting in many countries, the challenges in predicting WNV outbreaks are highlighted by the pattern of disease incidence in North America. Following introduction into the United States in 1999, the number of WNV cases increased steadily as the virus spread geographically. Cumulatively between 1999 and 2016 there have been over 46,000 symptomatic cases of WNV in the United States. Of these, 21,574 have resulted in neurologic disease, and over 2017 have been fatal.^4,5^ The largest number of reported WNV cases occurred in 2003, when almost 10,000 cases were documented in the US, resulting in 264 deaths.^6^ During the 2012 reporting season, the Centers for Disease Control reported the second highest number of WNV infections since the outbreak began, with 5674 total cases reported and 286 deaths, the highest yearly mortality in the U.S.^5^ Serious complications from WNV infection, which result from spread of the virus into the central nervous system, include meningitis, flaccid paralysis, and eventually death (reviewed in refs. ^7,8^). The continued geographic spread and consistent seasonal outbreaks of WNV coupled with the potential for increased disease severity highlight the need for development of effective vaccines.

Flaviviruses share a common genetic structure wherein the viral genome is translated as a single polypeptide that is co- and post-translationally processed to yield three structural and seven nonstructural proteins.^9^ The three viral structural proteins are the capsid (C) protein and the premembrane protein (prM), which is cleaved during virus maturation to yield the membrane (M) protein, and envelope (E) protein. The E protein contains the receptor binding and fusion functions of the virus, and X-ray crystal structures for the E protein of WNV and several other flaviviruses have been determined.^10,11^ The E protein can be divided into three distinct structural domains; DI, DII, and DIII. Antibodies to domains DII and DIII have been shown to neutralize the virus, and correlate with resolution of infection in preclinical models.^12^ As such, the WNV E-protein has been extensively evaluated as a vaccine candidate in both preclinical animal models and in the clinic (reviewed in refs. ^13,14^). WNV E protein antigen has been delivered as part of an inactivated virus,^15,16^ as a recombinant protein,^17–19^ as a DNA vaccine,^20^ as an RNA vaccine,^21^ and using various replicating and nonreplicating viral vectors.^14,22–25^ Live-attenuated vaccines for WNV have also been developed.^26,27^ Of the potential vaccine candidates, ChimeriVax-WN, the recombinant, live attenuated vaccine expressing WNV E protein in a YF17D backbone was tested in phase 2 clinical trials and induced high levels of neutralizing antibodies.^28,29^ A recombinant E subunit vaccine (WN-80E), absorbed to alum, also has been advanced into the clinic, but was found to induce low level neutralizing titers.^30^

Vaccine adjuvants are critical for the effective development of protective responses with many antigens. Ligands for Toll-Like receptors (TLR) including TLR 7/8 (Imiquimod, Resiquimod),^31^ TLR 9 (CpG),^32,33^ TLR 5 (Flagellin),^34^ and TLR 4^35–37^ have shown promise as components of immunostimulatory adjuvants in preclinical studies due to activation of the innate immune pathways triggered by natural infections. TLR4, TLR5 and TLR9 agonists have been specifically evaluated in combination with WNV E protein or domain III antigens, and have been shown to enhance protection and neutralizing antibody titers relative to alum-adjuvanted protein in mouse models.^3,38,39^

The use of combination adjuvants, which trigger complex and distinct innate immune pathways, has been shown in our previous work to provide enhanced protective responses. In the current study, we identify liposomal adjuvant formulations containing a second-generation synthetic lipid A (SLA) and the saponin molecule QS21 (SLA-LSQ) which induce increased virus neutralizing titers compared to other SLA formulations when combined with WN-80E. We have optimized the formulation, and have demonstrated synergy between SLA and QS21 components in the induction of neutralizing antibody responses. Using this optimized vaccine formulation, we demonstrate that a single immunization induces durable protection in mice and sterilizing immunity in hamsters. In addition, we examined the ability of SLA and QS21 to act synergistically, and report on the impact of this synergy on antibody diversity through repertoire analysis. Collectively, these studies define a promising adjuvant for use with recombinant flavivirus vaccine antigens, and suggest potential correlates of protection which may be evaluated in the context of other vaccine formulations.

## Results

### Identification and optimization of a combination liposomal adjuvant formulation containing SLA and QS21 results in induction of potent adaptive immune responses and neutralizing antibodies after a single immunization

**Fig. 1:**
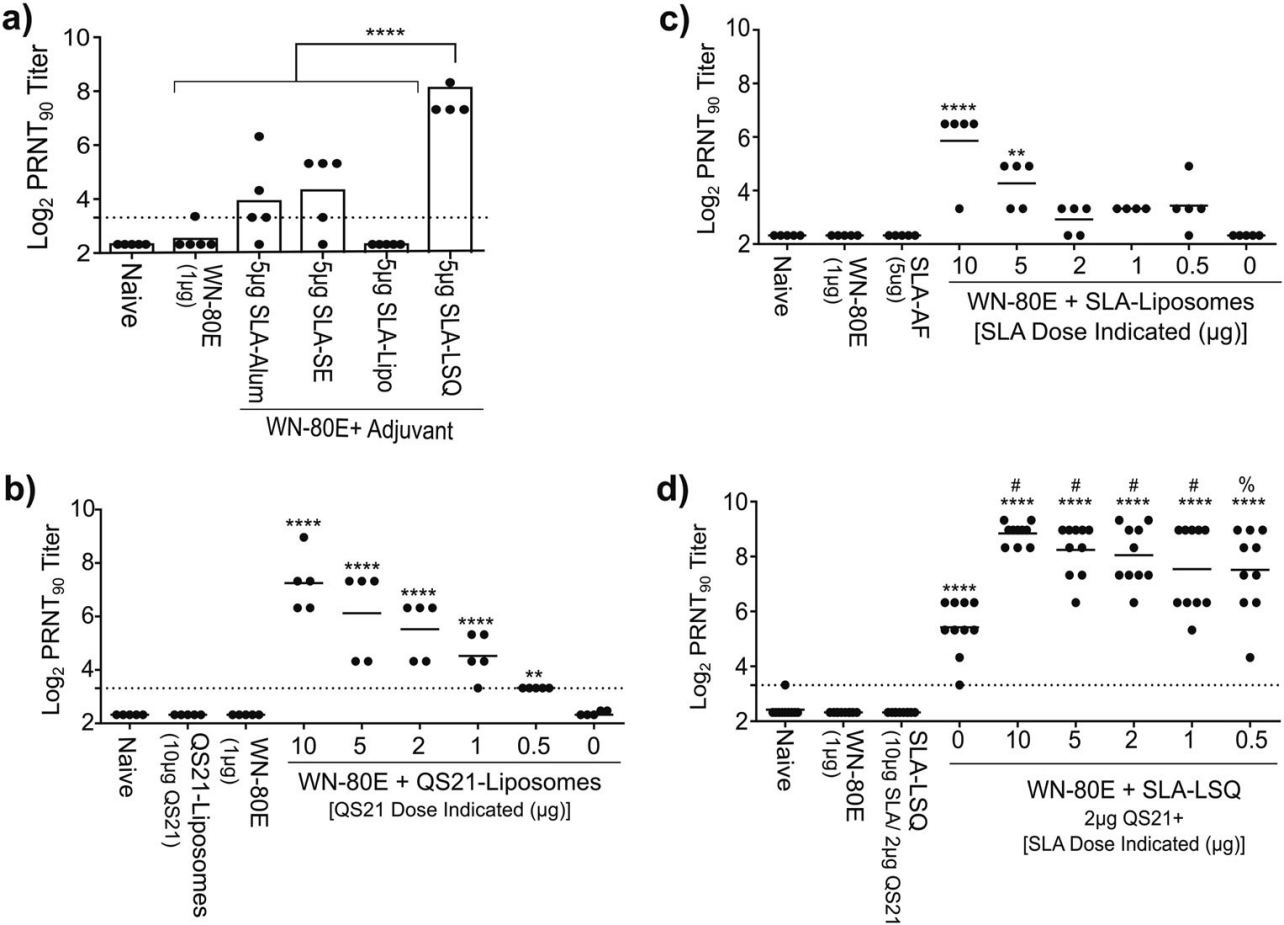
Identification and optimization of adjuvant components results in increased neutralizing antibody titers following a single immunization with WN-80E. In order to identify and optimize an SLA containing adjuvant formulation for WN-80E, we have examined the ability of different SLA containing adjuvant formulations to augment neutralizing antibody titers in combination with recombinant WN-80E protein. WN-80E was combined with SLA formulated in Alum (SLA-Alum), in a stable oil-in-water emulsion (SLA-SE), in a liposome (SLA-Lipo) and in liposomes containing QS21 (SLA-LSQ) **a**. All adjuvants contained 5 µg of SLA. Given elevated titers observed with SLA-LSQ, amounts of QS21 **b** and SLA **c** or both in combination (SLA-LSQ) **d** were optimized in liposomal adjuvants to maximize WNV neutralizing antibody responses, as measured by PRNT. Neutralizing antibodies were assessed 21 days post-immunization (*n* = 5/group, **a**–**c**, *n* = 10/group **d**). Bars indicate mean PRNT_90_ titers, dashed lines indicate the limit of detection of the assay. A dose-dependent increase in PRNT titer was observed following immunization with both QS21-Liposome and SLA-Liposome adjuvants. Significant increases relative to those observed in animals immunized with WN-80E alone are indicated (****p < 0.0001, ***p < 0.0005, **p < 0.01, *p < 0.05, one-way ANOVA). A liposomal adjuvant formulation containing 2 µg of QS21 and varied doses of SLA (SLA-LSQ) showed increased PRNT titers relative to WN-80E alone, and the inclusion of SLA resulted in a statistically significant increase in PRNT titer relative to animals immunized with WN-80E + QS21-Liposomes (^#^*p* < 0.01, ^%^*p* < 0.05, one-way ANOVA)

In previous work, we investigated the ability of the synthetic TLR-4 agonist SLA to enhance immunogenicity of a recombinant WNV E protein antigen (WN-80E), and found that SLA induced potent antiviral immunity when formulated with aluminum-oxyhydroxide/Alhydrogel (SLA-Alum), or in a stable emulsion containing squalene (SLA-SE).^3^ In the current study, we have compared a liposomal adjuvant formulation containing SLA (SLA-Liposomes), the saponin QS21 (QS21-Liposomes) or both SLA and QS21 (SLA-LSQ) to SLA-Alum and SLA-SE. Liposomal formulations contained the neutral lipid 1,2-Dioleoyl-sn-glycero-3-phosphocholine (DOPC). The dose of SLA tested in these studies (5 µg) was used in our previous studies, and has been used clinically in the case of SLA-SE. Following a single intramuscular immunization in 6−8-week-old C57Bl/6 mice (*n* = 5/group), we assayed serum for the presence of WNV neutralizing antibodies. Mice immunized with SLA-LSQ showed significantly increased WNV neutralizing antibody titers, as measured by a plaque-reduction neutralization test (PRNT_90_) relative to both WN-80E alone, and WN-80E in combination with SLA-SE or SLA-Alum (Fig. 1). Statistical significance was determined using one-way ANOVA relative to antigen alone (****p < 0.0001).

Due to the high level of neutralizing antibodies observed in our initial comparative studies, we undertook a systematic optimization of the SLA-LSQ formulation by examining the impact of both QS21 (Fig. 1b) and SLA (Fig. 1c) dose on neutralizing antibody induction when these agents are formulated in liposomes. Following a single intramuscular immunization combined with 1 µg of WN-80E (*n* = 5/group), we found that QS21 liposomes increased PRNT_90_ titer in a dose-dependent fashion; the increase was significant compared with antigen alone (Fig. 1b). Similarly, immunization of 6−8-week-old C57Bl/6 mice (*n* = 5/group) with WN-80E in combination with SLA liposomes also showed a dose-dependent increase in neutralizing antibodies, although in this case significant increases in PRNT_90_ titer were observed only at the higher 10 µg (*p* < 0.0001) or 5 µg doses (*p* < 0.0005) of SLA (Fig. 1c). In a final series of experiments, we examined the ability of optimized adjuvant formulations containing both SLA and QS21 (SLA-LSQ) to increase PRNT titers following a single intramuscular immunization (Fig. 1d, *n* = 10/group). For these studies, a fixed dose of 2 µg of QS21 was used. This dose was chosen based on previous preclinical and clinical experience with related adjuvant formulations, demonstrating adjuvanticity of formulations containing QS21 at this dose, and was confirmed by the pattern of neutralizing antibody induction observed following a boost-immunization with WN-80E + QS21-liposomes (Supplementary Figure S1). Mice immunized with SLA-LSQ formulations showed significant increases in PRNT_90_ titer compared to antigen alone, and the neutralizing antibody titers observed were higher than those measured following immunization with either QS21 or SLA alone (significance determined by one-way ANOVA). As with SLA liposomes, there was a dose dependence in overall antibody level observed following immunization with SLA-LSQ, and the titers observed in the presence of SLA-LSQ were increased significantly relative to the WN-80E + QS21-Liposome group (^#^*p* < 0.001, ^%^*p* < 0.05, one-way ANOVA).

**Fig. 2:**
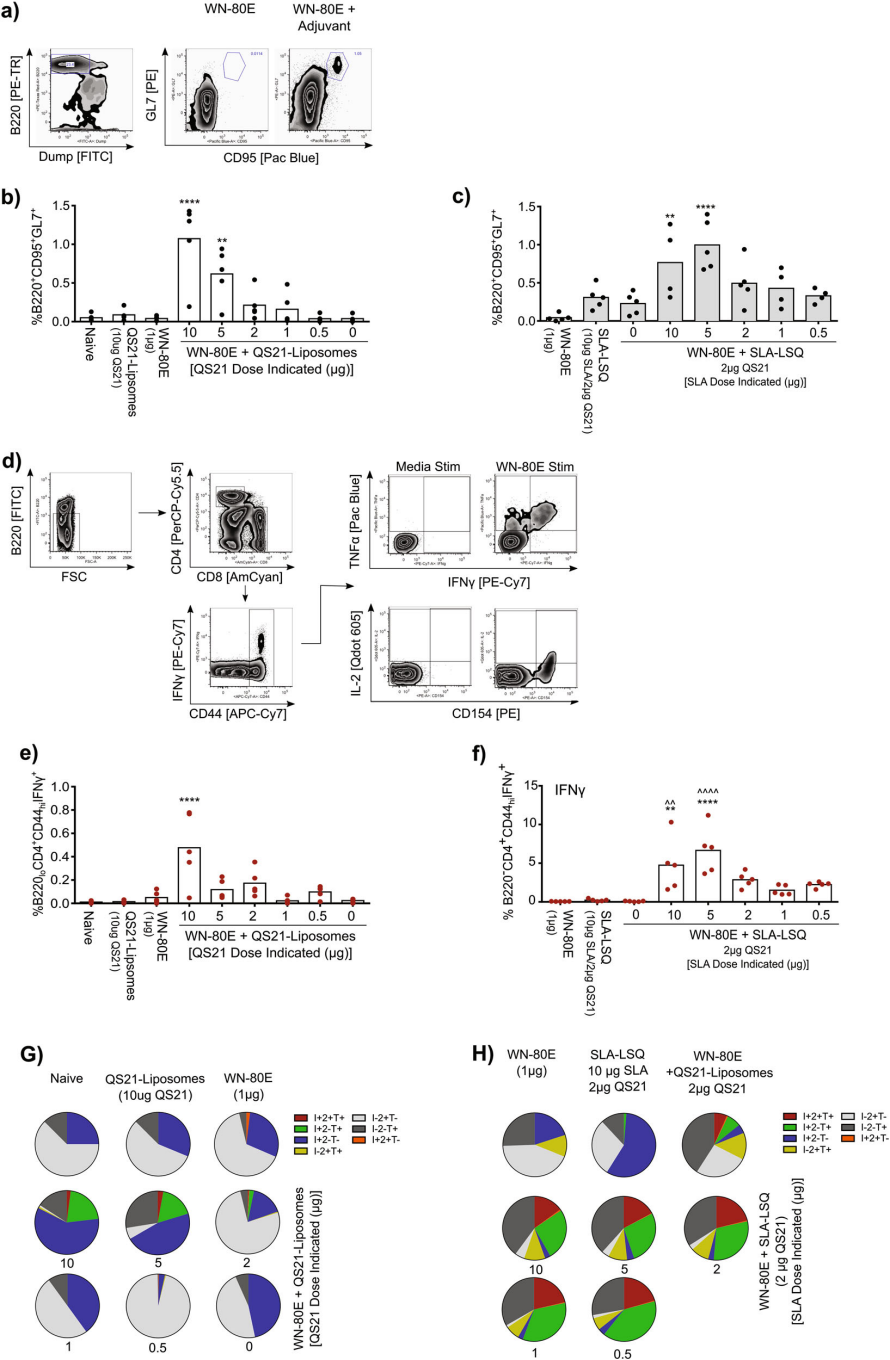
Induction of germinal center B cells and Th1 CD4+ T cells following immunization with WN-80E in combination with liposomal adjuvants. The ability of SLA-LSQ liposomal adjuvants to stimulate germinal center (GC) reactions **a**–**c** and CD4+ T-cell responses **d**–**h** 7 days following a prime immunization with 1 µg WN-80E (*n* = 5 mice/group) was investigated. Cytokine expression levels were determined for several characteristic Th1 cytokines including IFNγ (I), TNFα (T), and IL-2 (2). Inclusion of QS21 **b** or SLA and QS21 **c** induced significant increases in germinal center B cells in draining lymph nodes. Similarly, immunization with QS21-Liposome adjuvants induced low levels of IFNγ+ CD4+ T cells **e**, and very few Th1 CD4+ T cells (IFNγ+ IL-2+ TNFα+) only at high QS21 doses **g**. Addition of SLA-LSQ adjuvants (containing both QS21 and SLA) resulted in an increased number of IFNγ+ cells **f**, and many of these showed a canonical Th1 phenotype **h**. Significant increases relative to those observed in animals immunized with WN-80E alone (*) or WN-80E + QS21-Liposomes (^) are indicated (****/^^^^*p* < 0.0001, ***/^^^*p* < 0.0005, **/^^*p* < 0.01, */^*p* < 0.05, one-way ANOVA)

In addition to the analysis of humoral immunity, we examined the generation of cellular immunity following immunization with SLA-LSQ adjuvant formulations. We examined the induction of germinal center B cells in 6−8-week-old C57Bl/6 mice (*n* = 5/group) (CD95+ GL7+, Fig. 2a) in draining lymph nodes as well as induction of CD4+ T cells in the spleen (Fig. 2d). Intramuscular immunization of mice with 1 µg of WN-80E in combination with QS21-liposomal adjuvants resulted in significant increases in GC B cells (Fig. 2b), and relatively low levels of IFNγ+ T cells (Fig. 2e), which did not have a Th1 phenotype (IFNγ+ TNFα+ IL-2+, Fig. 2g). Intramuscular immunization with WN-80E + SLA-LSQ showed a similar increase in the levels of GC B cells, with significant increases observed at SLA doses of 10 and 5 µg (Fig. 2c). SLA-LSQ adjuvants also induced a significant increase in Th1 CD4+ T cells relative to antigen alone (*) or relative to antigen in combination with QS21-Liposomes (^, Fig. 2f). Many of the T cells induced following immunization with SLA-LSQ had a Th1 phenotype (Fig. 2h). In our previous work investigating novel adjuvants for recombinant influenza virus hemagglutinin (HA)-based vaccines, the induction of a potent Th1 CD4+ T-cell response, which results in directed class switching of antigen-specific antibodies to an IgG2c subtype in C57Bl/6 mice, was correlated with increased protection against both homologous and heterologous virus challenge.^40^ Here, the inclusion of 5 µg of SLA in combination with QS21 results in a directed Th1 CD4+ T-cell response that was absent following immunization with QS21 alone.

Collectively, these studies define an optimal SLA-LSQ adjuvant for use with WN-80E that contains 2 µg of QS21 and 5 µg of SLA. In addition to titration of QS21 and SLA, we have also examined the impact of adjuvant particle size on adjuvanticity. We find that, following intramuscular administration, all particle sizes tested demonstrate robust and similar immune enhancement as measured by the induction of virus-specific neutralizing antibodies (data not shown). We have also investigated the ability of SLA-LSQ to enhance WNV neutralizing titers following delivery by different routes, and find that in addition to intramuscular (IM) delivery, SLA-LSQ is effective in increasing titers when delivered via subcutaneous (SC) or intranasal (IN) routes (Supplementary Figure S2).

### WN-80E + SLA-LSQ stimulates production of long-lived functional neutralizing antibody responses to WNV in mice

**Fig. 3:**
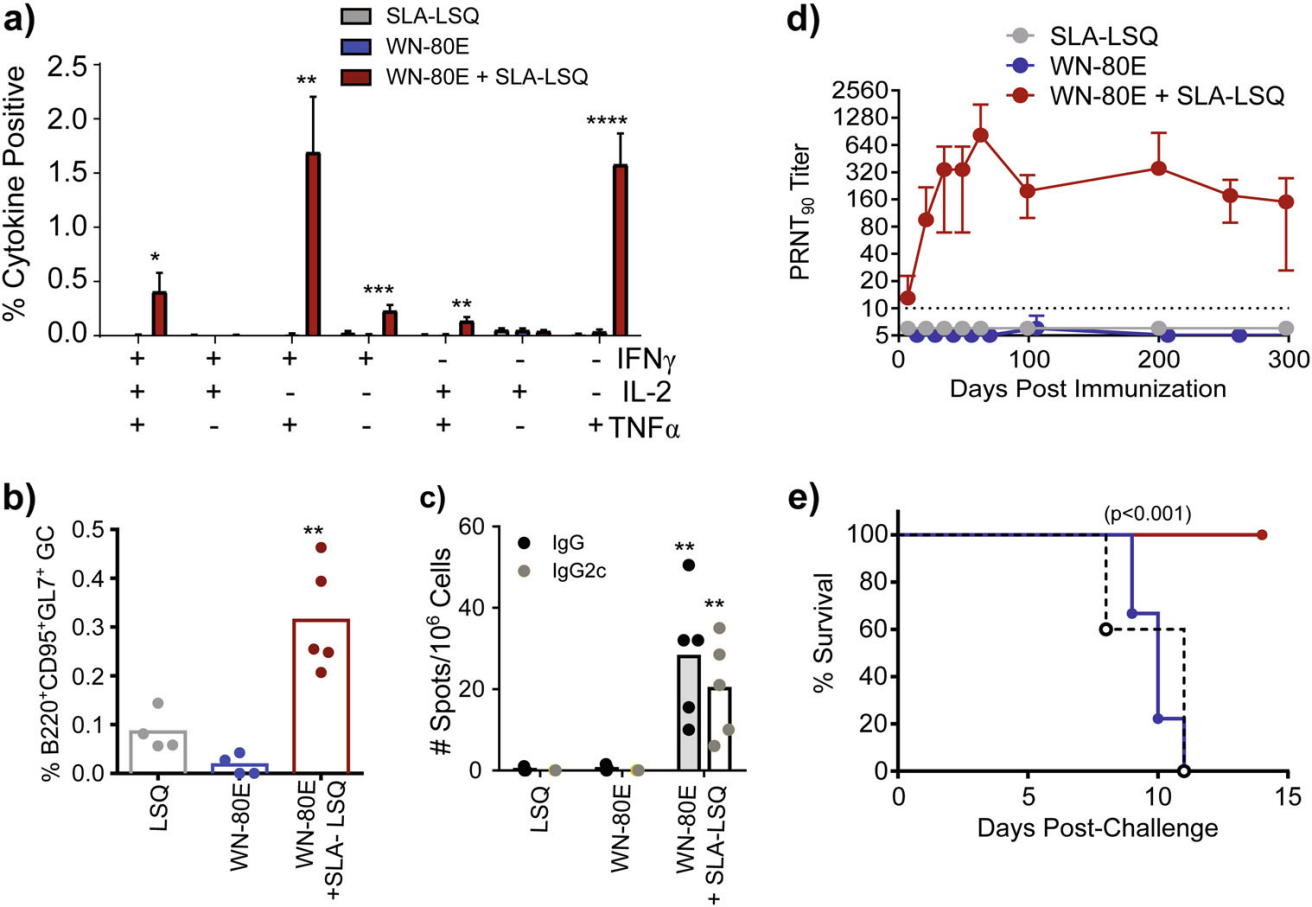
Optimized SLA-LSQ adjuvant formulations induce persistent functional immunity in mice. Following a single immunization of WN-80E (1 µg) with or without SLA-LSQ adjuvant (5 µg SLA/2 µg QS21), we investigated the longevity and functionality of antiviral antibody responses. Seven days post-immunization, and consistent with previous findings, we observe an adjuvant-dependent stimulation of CD4+ T cells **a** and germinal center B cells **b**. In addition, LSQ induced an increase in the number of WN-80E specific IgG+ antigen-secreting cells (ASC) in the bone marrow 21 days post-immunization. Consistent with induction of a Th1 CD4+ T-cell response, we find a significant increase in the number of IgG2c+ ASC at this timepoint **c**. Serum was periodically collected from immunized animals for up to 300 days. LSQ adjuvant stimulated a potent virus neutralizing response that peaked 63 days post-injection, and persisted for up to 300 days **d**. Three hundred days post-injection, all animals were challenged with 10^5^ PFU of WNV (NY385-99 strain). Animals immunized with SLA-LSQ adjuvanted vaccine showed 100% survival, while those immunized with WN-80E alone showed survival similar to naïve controls **e**. Bars in **a** represent mean percentages of cytokine-positive cells (*n* = 5/group), and error bars reflect standard deviation of the mean. Statistical significance was determined against WN-80E immunized animals by one-way ANOVA (*p < 0.05, **p < 0.005, ***p < 0.0005,****p < 0.0001)

In a subsequent study, we investigated the ability of SLA-LSQ to induce long-lived protective immunity (Fig. 3). Groups of 6−8-week-old C57Bl/6 mice (*n* = 20/group) were immunized with 1 µg of WN-80E alone or in combination with SLA-LSQ via the intramuscular route. Seven days post-immunization, mice were euthanized (*n* = 5/group) to assess the induction of cellular immunity. As described above, we observed adjuvant-dependent induction of Th1 CD4+ T cells in the spleen (IFNγ+ TNFα+ IL-2+, Fig. 3a), as well as significant numbers of IFNγ+ TNFα double-positive cells and IFNγ+ single cytokine-expressing cells (Fig. 3a). In addition, we observe induction of germinal centers in draining lymph nodes compared with animals vaccinated with antigen alone (Fig. 3b). Twenty-one days post-immunization, we examined bone marrow for the presence of antigen-specific long-lived antibody-secreting cells (ASC) by ELISPOT (*n* = 5/group), and found that animals immunized with WN-80E + SLA-LSQ show significantly higher IgG+ ASC relative to animals immunized with WN-80E alone. Consistent with the induction of CD4+ Th1 T cells, we found that many of the antigen-specific cells secrete IgG2c+ antibodies (Fig. 3c). In all cases, statistical significance was determined against WN-80E immunized animals by one-way ANOVA (*p < 0.05, **p < 0.005, ***p < 0.0005,****p < 0.0001). In a longitudinal study, we examined WNV-specific neutralizing antibody titers over a 300-day period. Mice (*n* = 5/group) immunized with 1 µg of WN-80E + SLA-LSQ had consistent levels of neutralizing antibody over 300 days, while those immunized with WN-80E alone had undetectable titers beginning 21 days post-immunization (Fig. 3d). To assess the function of these antibodies, we challenged animals at 300 days post-immunization (Fig. 3e). Animals immunized with WN-80E + SLA-LSQ showed 100% survival, while animals immunized with antigen alone showed similar survival to unimmunized controls. In a parallel study, we analyzed the longevity of antibody responses following two immunizations (Supplementary Figure S3). Similar to the results we obtained following a single immunization, two immunizations resulted in high titer neutralizing antibodies that persisted for 300 days. Collectively, these results highlight the ability of SLA-LSQ to induce long-lived functional WNV immunity when combined with a low dose of a WNV recombinant E protein antigen after a single immunization.

### Combination adjuvant formulations generate more diverse antibody responses following a single immunization

**Fig. 4:**
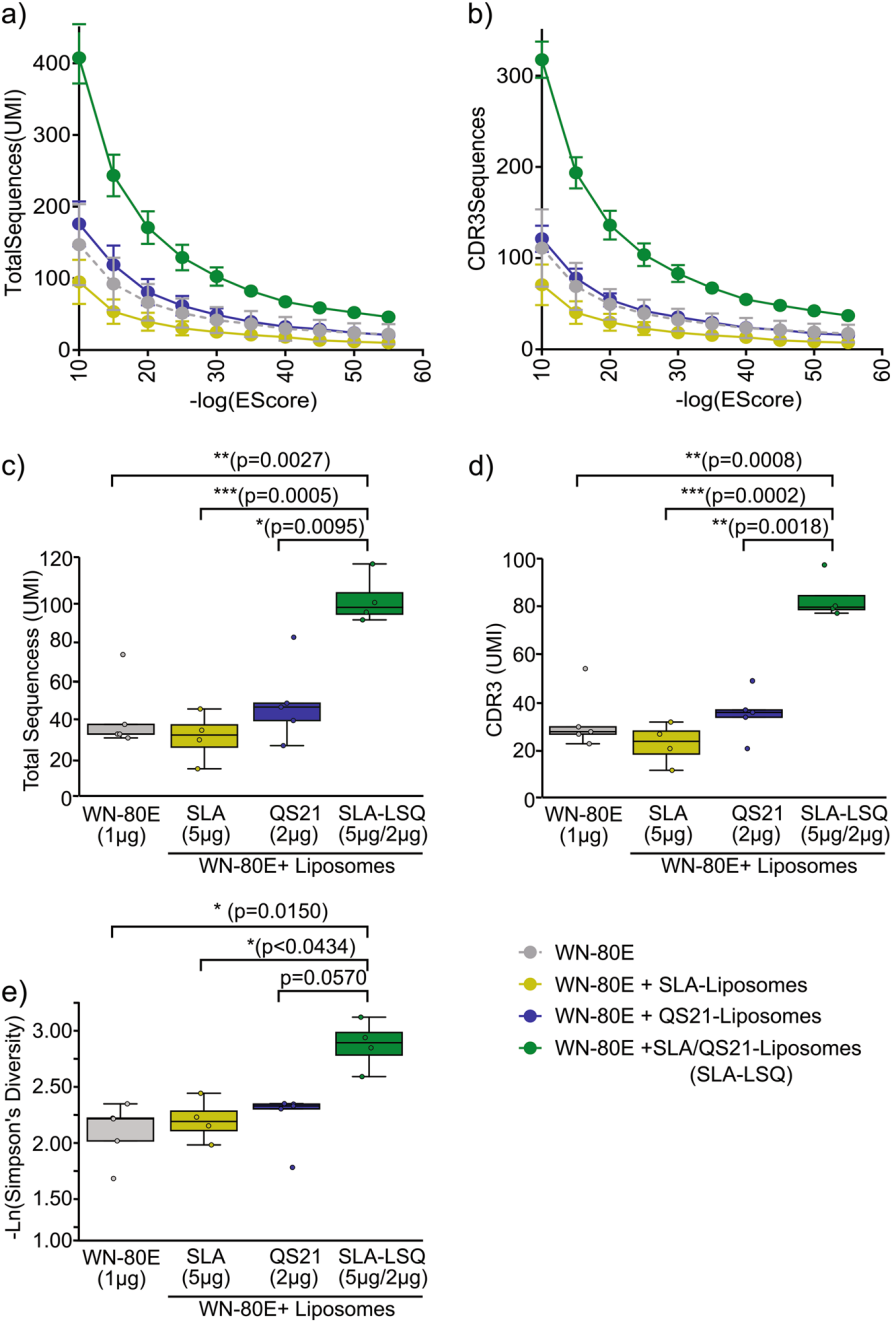
Protective adjuvant formulations induce a larger and more diverse pool of IgH V gene sequences following a single immunization. Mice (C57Bl/6) were immunized with 1 µg of WN-80E in combination with adjuvant formulations as indicated. Seven days following a prime immunization, bulk RNA was harvested from splenocytes. Antibody repertoire in splenocytes was assessed by HiSeq analysis of amplified CDR3 sequences as described previously.^41,45^ Sequences were quantified by identification of unique molecular identifiers (UMI), with specific sequences for each adjuvant determined by calculation of a significance score. The number of specific sequence counts **a** as well as the number of specific CDR3 sequences **b** was determined across a range of negative log e-score cutoff values. Using a cutoff value of 30, simulation analysis of both the total number of sequences **c** and the number of CDR3 sequences **d** was used to identify significant differences. In addition, the relative diversity of sequences in each specific pool defined by a cutoff value of 30 was determined by calculation of Simpson’s diversity index **e**. SLA-LSQ induced a significantly higher number of V-gene sequences and distinct CDR3 sequences, and the sequence pools were more diverse **e**. Significance was determined using nonparametric permutation analysis as described in Materials and Methods (*p < 0.05, **p < 0.005, ***p < 0.0005,****p < 0.0001)

Given the apparent synergy between SLA and QS21 in inducing potent virus-specific neutralizing antibodies, we examined the effects of SLA, QS21 or the combination SLA-LSQ adjuvant on antibody sequence diversity following a single immunization of mice. At 21 days following intramuscular immunization, RNA was prepared from splenocytes of immunized mice (*n* = 5/group), and antibody repertoire diversity assessed by deep-sequencing of antibody immunoglobulin heavy chain (IgH) variable (V) regions as previously described.^41^ The number of specific IgH V gene sequences induced by each adjuvant was determined across a range of significance scores, defined as the negative log of the e-score cutoff value, which indicate the probability of a given sequence being specific to that adjuvant group. SLA-LSQ induced a larger number of total IgH V-gene sequences (Fig. 4a) and distinct CDR3 sequences (Fig. 4b) across a range of cutoff values than either component alone. Over a broad range of values, the rank ordering of the groups remains identical, so the sequence sets defined at a significance score of 30 were used for further analysis. Simulation analysis of the enriched sequence pools showed that SLA-LSQ induced significantly higher unique (Fig. 4c) and CDR3 IgH V sequences (Fig. 4d) than SLA or QS21 liposomal adjuvants. The relative diversity of Ig V region sequences in each specific pool was determined by calculation of Simpson’s diversity index. SLA-LSQ resulted in a significant increase in diversity when compared to antigen alone, or to SLA containing liposomes. Comparison of SLA-LSQ to QS21 containing liposomes showed a trend toward significance, with a *p* value of 0.057 (Fig. 4e).

### SLA-LSQ reduces serum virus titer in Syrian golden hamsters following immunization with WN-80E

**Fig. 5:**
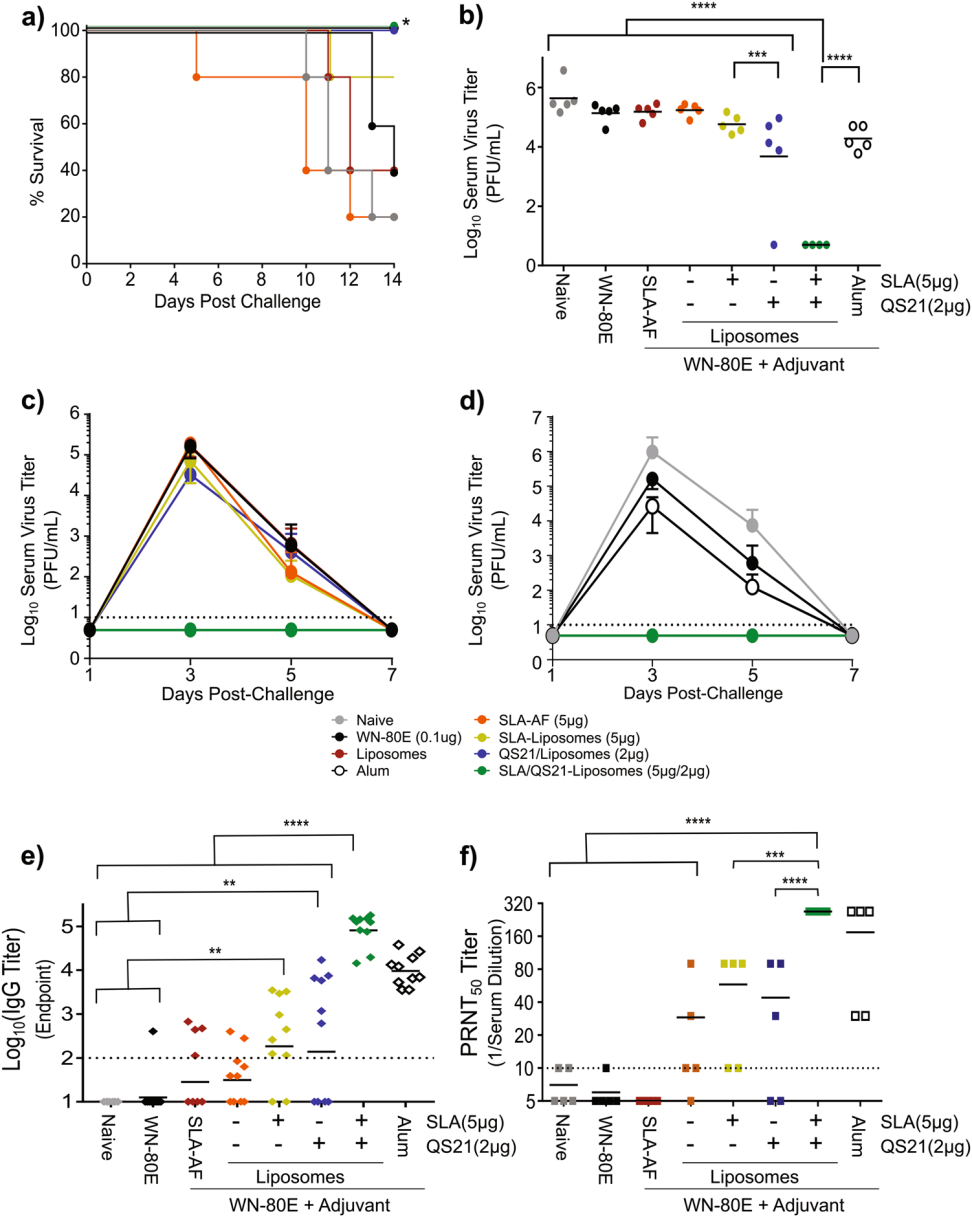
Optimized LSQ adjuvant formulations reduce viral load in hamsters following a single immunization. Syrian golden hamsters (*Mesocricetus auratus*, 4−5 weeks old, *n* = 10/group) were immunized with a low dose of WN-80E (100 ng) with or without liposomal adjuvants containing QS21 or SLA. Twenty-one days following a prime immunization, *n* = 5 animals were challenged with 10^5^ PFU of WNV via the intraperitoneal route. Animals were monitored for survival **a** for 14 days post-challenge. Serum was collected from all animals on alternate days from days 1−7 post-challenge. At 3 days post-challenge, which represents the peak of WNV replication, animals immunized with WN-80E + SLA-LSQ had no detectable virus titer, in contrast to other adjuvant formulations **b**. Examination of viral replication kinetics show that animals immunized with WN-80E + SLA-LSQ show no detectable titer at any timepoint **c**, **d**. Either QS21 or SLA alone failed to reduce titer relative to antigen alone **c**. Furthermore, at the reduced antigen doses tested here, SLA-LSQ was able to enhance protection relative to Alum **d**. Error bars **c**, **d** represent standard deviation from the mean. Prechallenge/post-immunization antigen binding **e**, and virus neutralizing **f**, antibody titers were measured 21 days post-immunization. Immunization with SLA-LSQ resulted in increased IgG endpoint titers, as well as significant increases in PRNT titers. (*p < 0.05, **p < 0.005***p < 0.0005, ****p < 0.0001, one-way ANOVA)

Given the persistent protection we observed with WN-80E + SLA-LSQ in mice (Fig. 3e), and the synergistic effects we observed in antibody repertoire diversity studies, we next investigated the effect of individual adjuvant components on protective capacity in Syrian hamsters. Briefly, hamsters (*n* = 10/group) were immunized with liposomal adjuvant formulations containing QS21 or SLA alone or with SLA-LSQ (Fig. 5). Twenty-one days post-immunization, animals (*n* = 5/group) were challenged with 10^5^ PFU of WNV (NY99 strain). This timepoint was chosen to mirror the antibody diversity analysis in mice. Immunization with SLA-LSQ,QS21-Lipsomes, or Alum resulted in 100% survival, which represents a significant increase relative to the 20% survival rate observed in animals immunized with WN-80E alone (significance determined using the Mantel−Cox test). Hamsters immunized with SLA-Liposomes showed 80% (4/5) survival, while immunization with liposomes alone or SLA-AF resulted in 40% survival that was similar to the 20% survival observed with WN-80E alone (Fig. 5a). To assess virus replication, sera were collected on D3 post-challenge to determine virus titers. The inclusion of adjuvants significantly reduced post-challenge virus titer relative to those observed in hamsters immunized with WN-80E alone (Fig. 5b). SLA-LSQ also resulted in significantly reduced titers relative to QS21-Liposomes or SLA-Liposomes, with no detectable virus present at this timepoint (Fig. 5b). In a subsequent study, we examined virus replication (*n* = 5 animals/group) over a 7-day period following a single immunization, and found that SLA-LSQ provided sterilizing protection; no virus was observed in any animal at any timepoint post-challenge, while significant replication was observed with QS21-Liposomes, SLA-Liposomes, or Alum, which has been previously tested in combination with WN-80E (Fig. 5c, d). In addition to virologic parameters, we have determined antigen-specific binding (Fig. 5e) and neutralizing (Fig. 5f) antibody titers immediately prior to challenge. Immunization with SLA-LSQ resulted in significant increases in antigen-specific binding IgG and neutralizing antibody titers relative to other nonprotective adjuvants, with significance determined by one-way ANOVA. We have also examined protective capacity of adjuvants combined with WN-80E after two immunizations (Supplementary Figure S4). This finding suggests that while both QS21 and SLA impart protective capacity alone, particularly after prime-boost immunizations, the presence of both components is critical for optimum adjuvant function and single-shot protection in this model.

**Fig. 6:**
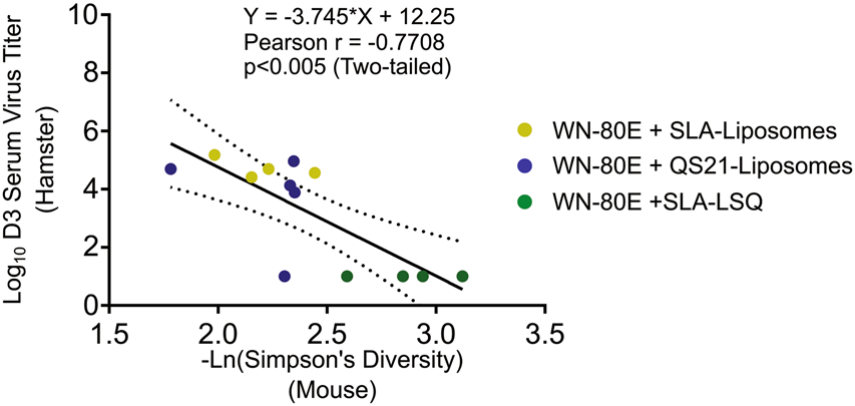
Correlation between increased antibody diversity in mice and virus titer reduction in Syrian hamsters. Correlation analysis was carried out on antibody diversity data in mice and D3 post-challenge serum virus titers in hamsters. Antibody diversity data in mice, presented as –Ln(Simpson’s Diversity Index), was compared to log-transformed serum virus titer in Syrian hamsters. Significant correlation (*p* = 0.0020, two-tailed) between the two parameters was observed following a Pearson analysis, with a correlation coefficient (*r*) of −0.7708, and an *r*^2^ value of 0.5942

Collectively, our data showed that protective adjuvant formulations in hamsters and mice generate diverse antibody responses. In order to determine whether a significant correlation between antibody diversity in mice and protection in hamsters exists, we have compared murine antibody diversity, graphed as −ln (Simpson’s Diversity Score), and log-transformed D3 serum virus titers in hamsters (Fig. 6). Pearson correlation analysis of these data showed a significant correlation between the two variables, with a correlation coefficient (*r*), or −0.708, and an *r*^2^ value of 0.5942. The significance value was 0.0020 (two-tailed *p* value), demonstrating a significant correlation between diversity and protection.

## Discussion

Despite continued circulation throughout the world, there are no approved vaccines or specific antiviral interventions for WNV in humans. In an effort to spur development of safe and effective vaccines, we combined WNV recombinant antigens with advanced adjuvant formulations containing the potent TLR4 agonist SLA or the saponin adjuvant, QS21. While both QS21 and SLA were able to improve neutralizing antibody titers to a recombinant WNV E protein antigen in liposomal formulations, we observe synergistic responses when both molecules are present; PRNT titers induced by SLA-LSQ are significantly increased relative to QS21 alone (Fig. 2). Examination of the cellular immune responses demonstrated that both QS21 and SLA stimulate germinal centers (Fig. 3b, c), but that only formulations containing SLA stimulate significant increases in antigen-specific Th1 CD4+ cells. Furthermore, the combination of these two components in SLA-LSQ leads to an increase in antibody diversity.

Previous work in our laboratory investigating the mechanism of action of combination adjuvant formulations has demonstrated synergistic roles for squalene containing emulsions (e.g. SE), that trigger the NLRP3-dependent inflammasome pathway, and TLR receptors, which stimulate NF-κb via a TRIF- and MyD88-dependent pathway, in the induction of Th1 CD4+ T cells^42^ as well as antigen-specific B cells.^43^ In the case of Th1 T-cell development, cell intrinsic and extrinsic production of IFNγ has been shown to promote CD69 activation on lymphocytes, which in turn may trap naïve lymphocytes in the draining lymph node alongside antigen-presenting B cells, ultimately driving a Th1 response. Consistent with this model, we observe a number of IFNγ+ T cells following SLA-LSQ in the spleen, and these cells may contribute to the development of a robust Th1 CD4+ T-cell response. In the case of B cells, CD169+ lymph node resident macrophages are implicated in the early uptake of adjuvant formulations and production of IL-18, ultimately leading to the expansion of B-cell responses. This expansion ultimately results in generation of a more robust B-cell response that includes pre-plasmablasts responsible for early production of antigen-specific antibodies. QS21 has recently been shown to trigger components of the inflammasome,^44^ suggesting that SLA-LSQ may act via a similar synergistic mechanism. The contribution of different lymph node resident cells and other migratory cell types including dendritic cells and other APCs will be assessed in future studies. Collectively, these previous studies provide a potential mechanism of action consistent with the enhanced cellular and humoral immune responses that we observe with SLA-LSQ.

In addition to monitoring the induction of virus neutralizing antibodies, we examined the impact of adjuvanted vaccination on overall antibody diversity. This work builds on our previous findings showing that antibody sequence diversity is increased following immunization with TLR agonist adjuvant formulations,^45^ but in a model system where antibodies are believed to be the primary correlate of protection. Similar to the findings we observe in induction of WNV neutralizing antibodies, we find that SLA and QS21 act synergistically to increase antibody diversity, as measured by application of Simpson’s diversity index. Both QS21-liposomes and SLA-liposomes showed a modest increase in the number of unique IgH V-gene sequences (Fig. 4c) and CDR3 sequence (Fig. 4d) relative to immunization with WN-80E alone that was not significant, whereas SLA-LSQ-based adjuvants showed a pronounced and significant increase in the number of sequences that is not additive. The total number of sequences in SLA-LSQ is greater than the sum of sequences in the SLA-liposomes and QS21-liposomes, and examination of the sequences in each pool showed specific sequences in the SLA-LSQ immunized animals that were not present in the QS21-Liposome or SLA-Liposome immunized animals. Furthermore, there was a trend toward increased diversity of sequences observed in the SLA-LSQ immunized animals compared to SLA-liposomes or QS21-liposomes. We show a significant correlation between this increase in diversity, measured in mice, with protective capacity in the Syrian hamster model. While preliminary, these findings suggest a novel antibody diversity metric which may be used to preclinically evaluate adjuvant formulations capable of stimulating protective immunity. Our previous studies have shown that GLA-SE, another inflammasome/TLR-4-activating adjuvant, can induce broadly protective antibody responses, capable of neutralizing heterologous virus strains.^40^ Given this phenotype, and the observed increases in diversity, it is possible that SLA-LSQ will induce antibody responses capable of neutralizing both homologous and heterologous flavivirus antigens. This possibility will be tested in future studies. Additional work is also planned to determine whether the correlation between antibody diversity and protective antibody responses is generally applicable to other adjuvant formulations and adjuvant−antigen combinations, and will investigate the B-cell compartments which give rise to the observed diversity. Additionally, we plan to screen recovered antibody gene sequences by generating recombinant antibodies to determine whether adjuvants may be used as a tool to reveal rare or unique V-gene sequences and usage, which in turn may allow isolation of IgV-H gene sequences with unique protective capacity. This approach has been used previously to identify broadly reactive influenza antibodies.

Collectively, the potent protective capacity we observe with SLA-LSQ is consistent with a model wherein induction of a diverse antibody response, driven by expansion of germinal center B cells and induction of a Th1 CD4+ T-cell response, results in increased protective capacity of a flaviviral antigen. Additional experiments are planned to extend this finding to other members of the flavivirus family, and to test SLA-LSQ in combination with multimeric flavivirus VLP-based antigens which are capable of stimulating antibodies to the quaternary E-protein epitopes shown to be critical for neutralization of DENV.^46–48^ Should these studies demonstrate a broad applicability of SLA-LSQ in the enhancement of flaviviral immunity, we believe that the adjuvant formulation described here may be directly advanced to the clinic. A similar adjuvant formulation containing 2 µg of QS21 and 5 µg of the related TLR-4 agonist glucopyranosyl lipid adjuvant (GLA) has been tested in the clinic with a recombinant Leishmania antigen, and was observed to be safe, and to enhance immunity. As such, the adjuvant formulation identified here represents a promising agent for the improvement of immunogenicity and for clinical advancement of vaccines for WNV or other flaviviruses (e.g. Zika) as well as additional emerging disease vaccines from other virus families.

## Materials and methods

### Virus stocks and vaccines

Stocks of WNV (NY99 strain) were prepared from infected Vero cells (CCL-81, ATCC). Briefly, confluent cells were inoculated with WNV at an MOI of 0.1. Virus growth medium (MEM supplemented with 5% fetal bovine serum) was added to the flask after the virus was adsorbed onto drained monolayers for 60 min. Cells were examined daily following infection, and supernatant was harvested when cytopathic effect was evident throughout the culture. Decanted medium from the infected cells was clarified by centrifugation at 5000 × *g* for 10 min. Clarified supernatant was supplemented with additional FBS to a concentration of 15%. Virus was aliquoted and stored at −80 °C. Thawed stocks were titrated by plaque assay with titers of virus stocks typically 10^8^ pfu/mL.

### Proteins and adjuvants

The WN-80E protein utilized in these studies was provided by Hawaii Biotech, and has been previously described.^17^ Briefly, the protein is a carboxy-truncated WNV E-protein which is produced in Drosophila S2 cells, and purified using an antibody affinity-based purification method. Protein was provided in PBS, and stored at −80 °C until use.

SLA, a synthetic lipid adjuvant, is a derivative of GLA and has been previously described.^49^ QS21 was obtained through in-house purification of Quil A (Brenntag Biosector), or purchased from Desert King (San Diego, CA). QS21 purity was assessed in each lot via a high-performance liquid chromatography-based method established at IDRI and qualified for cGMP release. Liposomes were generally manufactured by microfluidization until a liposome diameter of ~80 nm was achieved.

### Immunogenicity studies

All animal work conducted in this study, including immunogenicity and WNV challenge studies, was specifically approved by the Infectious Disease Research Institute (IDRI) Institutional Animal Care and Use Committee (IACUC). All mice and hamsters were maintained for the duration of the study in specific pathogen-free conditions.

For murine immunogenicity studies, 6−8-week-old female C57Bl/6 mice were immunized via the intramuscular route in a final volume of 100 μL/immunization (50 μL delivered to each leg) at 0 (prime) and 21 (boost) days. Seven days following each immunization serum, spleen and inguinal lymph nodes were collected for analysis. Twenty-one days following each injection, additional serum and bone marrow were collected for analysis of WNV-specific antibody titers and for ELISPOT analysis.

### Challenge studies

For murine challenge studies, 6−8-week-old female C57Bl/6 mice were challenged with 10^5^ plaque forming units (PFU) of WNV via intraperitoneal injection of virus in 0.25 mL total volume. Following challenge, all animals were observed daily for signs of virus-induced morbidity and mortality. Seventy-two hours following challenge, peripheral blood was obtained from all animals via retro-orbital bleed to determine serum virus titers. For survival studies, and per institutional IACUC guidelines, animals showing overt neurological symptoms accompanied by weight loss following challenge were euthanized.

For hamster challenge studies, 4−5-week-old male Syrian golden hamsters (Mesocricetus auratus) were challenged with 10^5^ PFU of WNV via the intraperitoneal route in 0.25 mL total volume. Seventy-two hours following challenge, blood (approximately 1 mL/animal) was collected from all animals via cardiac puncture. This timepoint was chosen as it is near the peak of serum virus titer observed following challenge in this species. For 14 days following challenge, animals were monitored daily for weight loss and for signs of virus-induced morbidity. Per institutional IACUC guidelines, animals showing overt neurological symptoms accompanied by weight loss following challenge were euthanized.

### Plaque assay

Serial tenfold dilutions of serum were prepared in BA-1 medium (M-199 salts, 1.0% bovine serum albumin, 350 mg/L sodium bicarbonate, 100 units/mL penicillin, 100 mg/L streptomycin, and 1.0 mg/L amphotericin in 0.05 M Tris [hydroxymethyl aminomethane], pH 7.6) were prepared in 96-well plates (Corning). Diluted samples were added to six-well (Corning) plates seeded 24 h prior with 10^6^ Vero cells/well, and incubated for 60 min with shaking at 15-min intervals to ensure even virus distribution. Wells were overlaid with a 0.5% agarose (Seakem) solution and incubated at 37 °C for 48 h to allow plaque formation. Following incubation, cells were stained with crystal violet to visualize and enumerate plaques.

### Plaque-reduction neutralization titer (PRNT) assay

Sera from immunized mice were inactivated by incubation at 56 °C for 30 min. Inactivated sera were serially diluted twofold in BA-1 medium in a 96-well plate (Corning) beginning with a 1:5 dilution in a total volume of 100 µL. Following serum dilution, 100 µL of diluted virus (200 pfu) was added to all serum samples. Virus:serum mixtures were incubated at 37 °C for 60 min. Following incubation, virus in all samples was titrated using standard plaque assay techniques. Briefly, virus−serum mixtures were incubated with Vero cell monolayers (200 µL/well) at 37 °C for 60 min with rocking to distribute the medium every 15 min. Wells were overlaid with 0.5% agarose and incubated for 3 days at 37 °C in a CO_2_ incubator. Following this incubation, plaques were stained with crystal violet prior enumeration. Negative (media only) and convalescent post-challenge murine serum were included in each assay. Neutralizing antibody titers are presented as the highest total serum dilution capable of reducing the number of plaques by 90% compared to a virus only control (PRNT_90_).

### Enzyme-linked immunosorbent assay (ELISA)

WN-80E-specific IgG endpoint titers in Hamsters were determined 1 day prior to challenge. High binding polystyrene 96-well plates were coated with WN-80E (2 µg/mL) in 0.1 M bicarbonate coating buffer for 2.5 h at room temperature. Plates were washed three times with 0.1% PBS–Tween 20 before and after a 2-h blocking incubation with 0.05% PBS–Tween 20 + 1% BSA at room temperature. Hamster sera was serially diluted in 0.05% PBS–Tween 20 + 0.1% BSA, added to plates, incubated overnight at 4 °C, and washed five times. Plates were incubated for 1 h with anti-hamster IgG HRP-conjugated secondary antibody (Southern Biotechnologies) with shaking. Following five washes, plates were developed by the addition of SureBlue tetramethylbenzidine substrate (Kirkegaard & Perry Laboratories) using a Nanoscreen robot. The enzymatic reaction was stopped with 1 N H_2_SO4 using a Multipette Sagian robot. Plates were read at 450−570 nm using the Synergy ELISA plate reader (Biotek) and Gen5 software.

### Intracellular cytokine staining

In order to quantify vaccine-specific T-cell responses, splenocytes were isolated from five mice per group 7 days post-immunization. Red blood cells were lysed using Red Blood Cell Lysis Buffer (eBioscience) and resuspended in cRPMI 1640 (10% FBS, 1% penicillin/streptomycin; 0.1% 2-Mercaptoethanol). Cells were plated at 10^7^ cells/well in 96-well plates and were stimulated for 2 h with media or WN-80E Antigen (10 µg/mL) at 37 °C. At *t* = 2 h, 1:50 GolgiPlug (BD Biosciences) was added and the cells were incubated for an additional 8 h at 37 °C to allow cytokine production. Cells were washed and surface stained with fluorochrome-labeled antibodies (1:100 dilution in 1% BSA−PBS) to CD4 (clone RM4-5), CD8 (clone 53-6. 7), CD44 (clone IM7), and B220 (RA3-6B2) (BioLegend and eBioscience) in the presence of anti-CD16/32 (clone 93) for 15 min in the dark at room temperature. Cells were fixed and permeabilized with Cytofix/Cytoperm (BD Biosciences) for 30 min at room temperature in the dark. Cells were washed with Perm/Wash (BD Biosciences) and stained for 15 min with fluorochrome-labeled antibodies to detect intracellular cytokines as follows: IFN-γ (clone XMG-1.2), IL-2 (JES6-5H4), TNF (MP6-XT22), IL-5 (clone: TRFK5), and IL-10 (clone: JES5-16E3) (BioLegend and eBioscience). Cells were washed, resuspended in 1% BSA−PBS and filtered using a 30−40 µm PP/PE 96-filter plate (Pall Corp). Up to 10^6^ events were collected on a four-laser LSR Fortessa flow cytometer (BD Biosciences). Data were analyzed with FlowJo (Treestar).

### B-cell quantification

Seven days following immunization, inguinal lymph nodes were isolated from five animals per group. Cells were resuspended in cRPMI 1640 (10% FBS, 1% penicillin/streptomycin; 1:1000 2-Mercaptoethanol) and plated at 10^7^ cells/well in 96-well plates. Cells were surface stained in staining buffer (1% FBS, 1:250 EDTA, PBS) with fluorochrome-labeled antibodies (1:200) to CD138 (clone281-2), GL7 (clone GL7), CD95 (clone Jo2), IgM (clone II/41), CD19 (clone 1D3 or 6D5), IgD (clone 11-26 c.2a), CD38 (clone 90), and 1:100 CD16/32 (clone 93) for 15 min in the dark at 4 °C. Non-B-cell lineage cells were excluded by staining (1:200) and gating for Ly6G (clone 1A8), CD11b (clone M1/70), CD11c (clone N418), F4/80 (clone BM8), Ter119 (clone TER-119), and Thy1.2 (clone 53-2.1) hi populations. Cells were fixed and permeabilized with Cytofix/Cytoperm (BD Biosciences) for 20 min at room temperature in the dark and washed with Perm/Wash (BD Biosciences). IgG subtype staining was carried out for IgG1 (clone RMG1-1) and biotinylated-IgG2a,b,c (clone 5.7). IgG2a,b,c was detected by addition of streptavidin (1:500) for 15 min at 4 °C in the dark. Cells were resuspended and filtered in staining buffer using a 30−40 µm PP/PE 96-filter plate (Pall Corp). Up to 10^6^ events were collected on a four-laser LSR Fortessa flow cytometer (BD Biosciences). Data were analyzed with FlowJo (Treestar).

### Antibody repertoire analysis

Preparation, sequencing and data analysis were performed as described with the following modifications.^41^ In addition to the combined barcode UMI sequence in the cDNA primer, an additional six base barcode was added to the 3′ end of the upstream PCR primer, allowing samples from each individual to be tagged with a unique barcode pair. Sequencing of pooled barcoded samples was performed with a 2 × 250 HISEQ analysis, yielding 101,692,094 total paired end reads of which a total of 41,287,384 had designated barcode pairs. All paired sequences with identifiable primer sets and bar codes were grouped and assembled using a method designed to align sequences representing a single molecular species of IgH V-genes, and treatment group e-scores for each assembly were calculated using the formula described.^45^ Specifically, the e-score for each assembly of sequences with regard to a treatment group was calculated by summing UMI counts from all individuals in the group vs. individuals in the control group at day 7 and day 21 post-immunization. If the negative log value of the group’s e-score was greater than or equal to the cutoff value, then that assembly is considered specific to that treatment group. A significance score cutoff value of 30 was used for the diversity calculations in Fig. 4, which means that given a random distribution cDNA events between groups, the probability of seeing the observed UMI counts or greater in the experimental group as compared to the control group is less than 1×10^−30^.

Simpson’s diversity index was calculated as described.^50^ Since the measure yields small numbers (indicating greater diversity) with large datasets, the negative log of the Simpson’s diversity index is reported (larger is more diverse). We used nonparametric permutation tests to evaluate the difference in diversity between mice in two adjuvant groups. The test was performed by randomly shuffling mice from two groups, then recomputing diversity for each shuffled group, and comparing the diversity in each. This was repeated 10,000 times and the significance of the observed data was demonstrated by the fraction of times that a shuffled dataset produced a result that was as extreme as the observed difference in diversity (i.e. the *p* value).

### Data availability

The datasets generated during and/or analyzed during the current study are available from the corresponding author on reasonable request.

## Electronic supplementary material

Supplementary Information

## Electronic supplementary material

**Supplementary information** accompanies the paper on the *npj Vaccines* website (10.1038/s41541-018-0077-1).
