# Supplementary material for: A combination of TLR-4 agonist and saponin adjuvants increases antibody diversity and protective efficacy of a recombinant West Nile Virus antigen
Source: NPJ Vaccines. 2018 Sep 26;3:39. doi: 10.1038/s41541-018-0077-1 (PMC6158298; doi:10.1038/s41541-018-0077-1)
Supplement: Supplementary file 1 — Supplementary Information [file 41541_2018_77_MOESM1_ESM.pdf]

TITLE: A Combination of TLR-4 Agonist and Saponin Adjuvants Increases Antibody Diversity and Protective Efficacy of A Recombinant West Nile Virus Antigen

Running Title: Development of an SLA-QS21 Adjuvant for Flavivirus Vaccines

Neal Van Hoeven[1,3#], Steven Wiley[2], Emily Gage[1,3], Andrew Fiore-Gartland[4], Brian Granger[1], Sean Gray[5], Christopher Fox[1], David E. Clements[6], D. Elliot Parks[6], Scott Winram[7], Dan T. Stinchcomb[1], Steven G. Reed[1], Rhea N. Coler[1,3,5].

Supplementary Information:

Supplementary Figure S1

Supplementary Figure S2

Supplementary Figure S3

Supplementary Figure S4

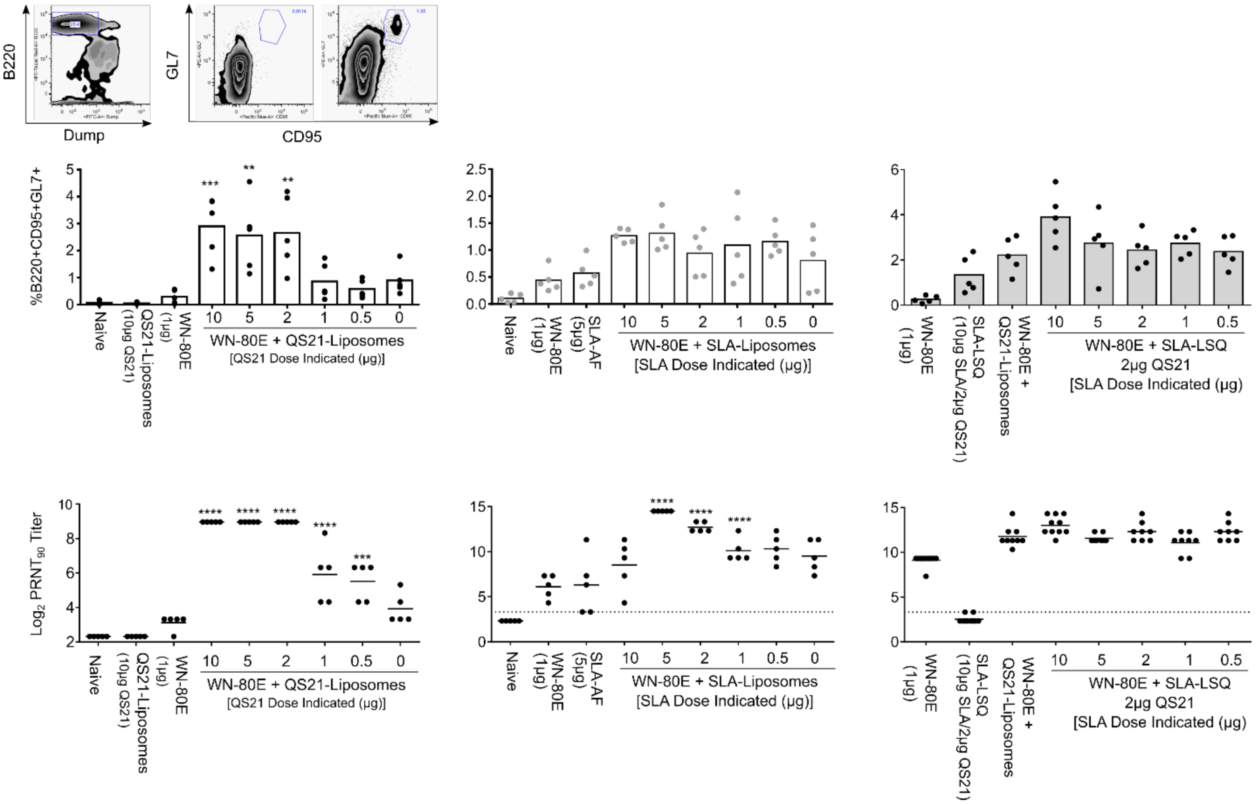

Supplementary Figure S1: Induction of Germinal Center B-Cells and Neutralizing Antibody Titers Following Boost Immunization.

Following an intramuscular boost immunization of WN-80E (1 μg) with SLA or QS21 containing adjuvant formulations, we investigated the induction of germinal center (GC) B-cells in draining lymph nodes at D7 post-immunization and serum antibody titers at 21 days in 6-8 week old C57Bl/6 mice (5-10/group). QS21 doses between 2 μg and 10 μg resulted in induction of GC B-cells and neutralizing antibody titers. Similarly, inclusion of SLA resulted in GC B-cells and neutralizing antibody titers. The combination of SLA and QS21 (SLA-LSQ) resulted in neutralizing antibody titers across a broader range of SLA titers. Significance is indicated relative to titers observed following immunization with WN-80E alone (*p<0.05, **p<0.005***p<0.0005, ****p<0.0001, One-way ANOVA).

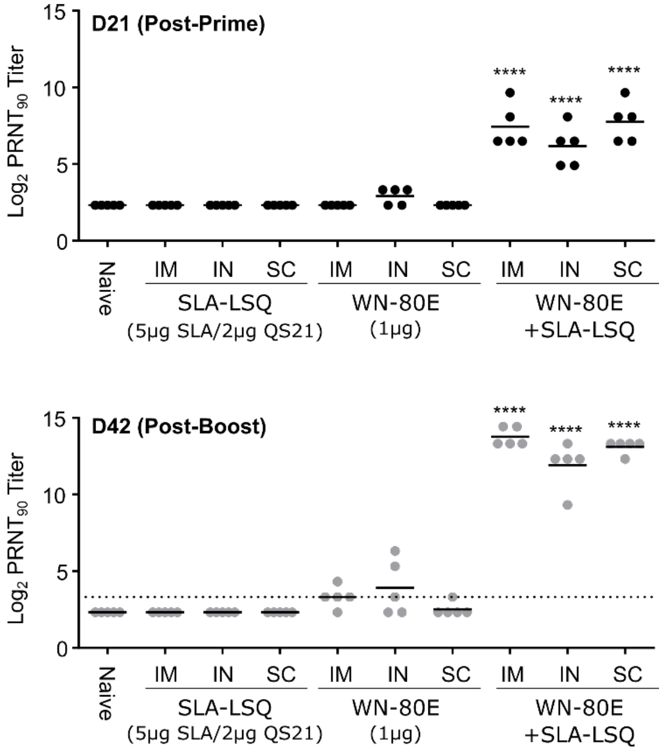

Supplementary Figure 2: Optimized Adjuvant Formulations Stimulate High Titer Neutralizing Antibodies by Different Immunization Routes.

6-8 week old C57Bl/6 mice (n=5 mice/group) were immunized twice with WN-80E alone or in combination with an optimized SLA-LSQ adjuvant formulation via the intra-muscular (IM), intra-nasal (IN) or sub-cutaneous (SC) routes. 21 days following each immunization, serum was collected to determine virus neutralizing titers. For all routes tested, inclusion of adjuvant resulted in a statistically significant increase in virus neutralizing titer relative to antigen alone delivered by the same route (****$p<0.0001$, One-way ANOVA). Delivery of optimized vaccines resulted in similar neutralizing antibody titers regardless of the route of immunization.

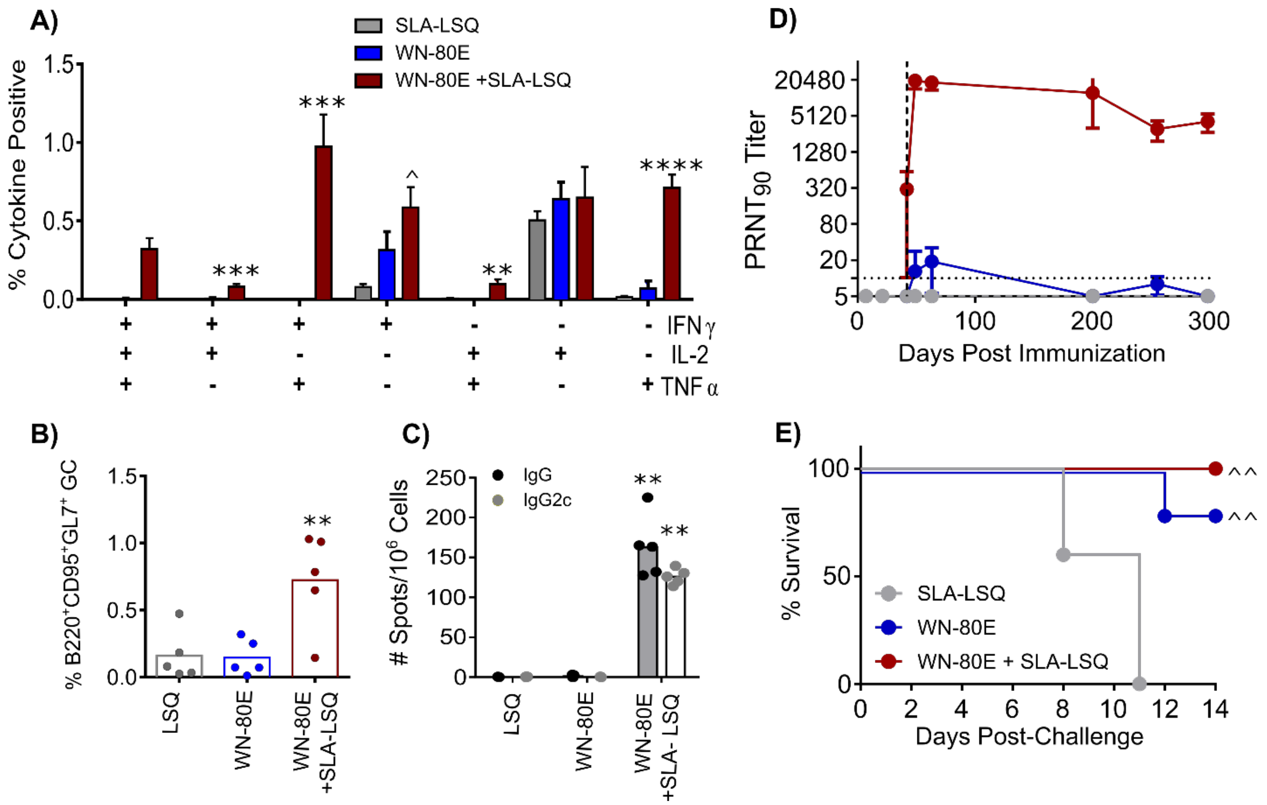

Supplementary Figure S3: Optimized LSQ adjuvant formulations induce long-lived functional immunity Following Boost Immunizations in mice.

Following a boost immunization of WN-80E (1µg) with or without SLA-LSQ adjuvant (n=20/group), we investigated the durability and functionality of antiviral antibody responses. 7 days post-boost immunization, we observe adjuvant dependent stimulation of CD4+ T-cells (A, n=5/group)) and germinal center B-cells (B, n=5/group)). In addition, SLA-LSQ induced an increase in the number of WN-80E specific IgG+ antigen secreting cells (ASC) in the bone marrow relative to those observed following a single immunization (C, n=5/group). Serum antibodies, as determined by PRNT assay, persisted for up to 300 days (D, n=5/group). 300 Days post injection, all animals were challenged with 10^5 PFU of WNV (NY385-99 strain) (E). Significance in A-C is indicated relative to titers observed following immunization with WN-80E alone (*p<0.05, **p<0.005***p<0.0005, ****p<0.0001, One-way ANOVA). Significance in E is determined by Mantel-Cox log rank test (^^ p<0.005).

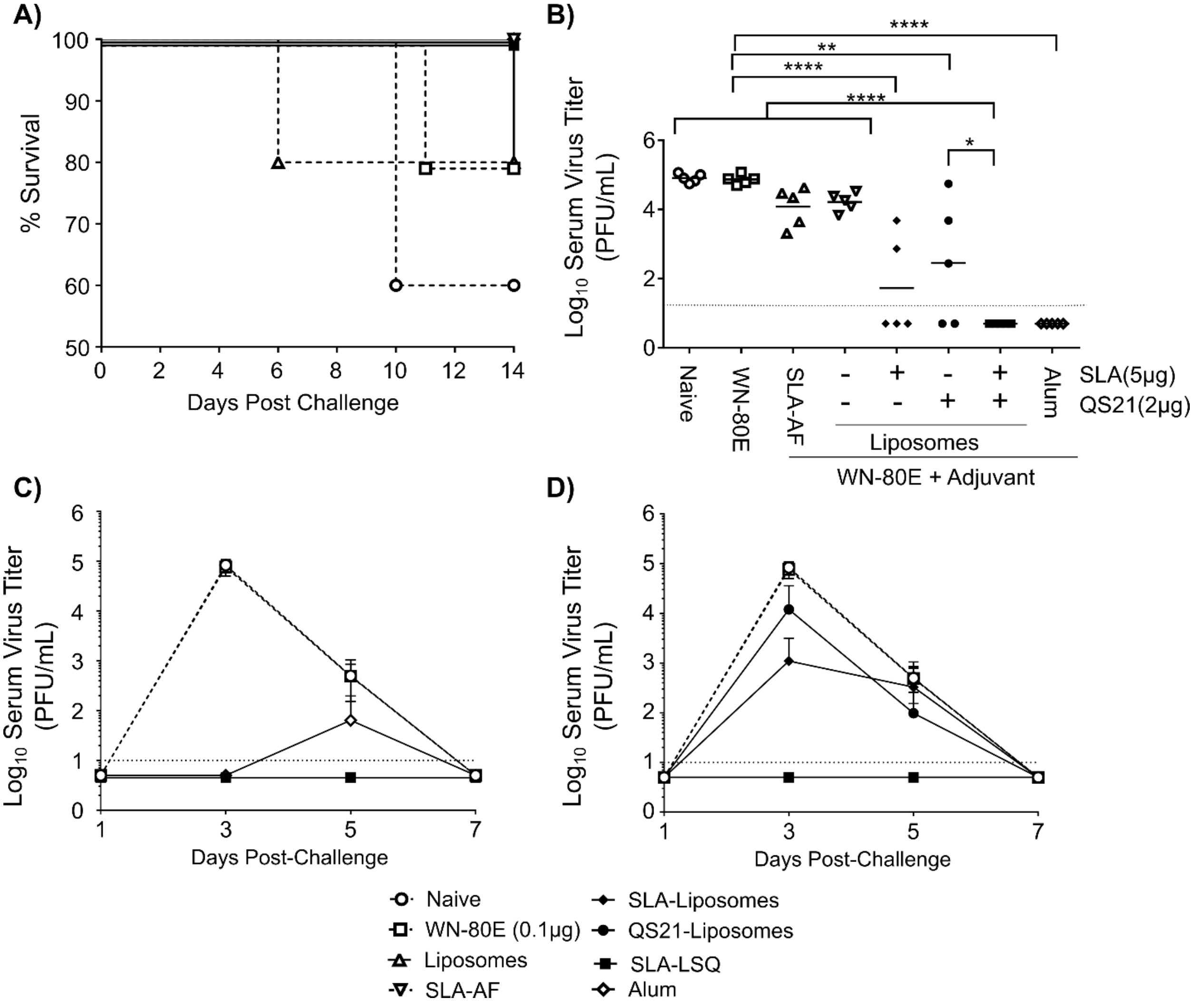

Supplementary Figure S4: Hamster Challenge Following Prime-Boost Immunization with WN-80E.

Syrian golden hamsters (Mesocricetus auratus, 4-5 weeks old, n=5/group) were immunized twice (D0, D28) with a low dose of WN-80E (100ng) with or without liposomal adjuvants containing QS21 or SLA. Twenty-one days following a boost immunization, animals were challenged with 10^5PFU of WNV via the intra-peritoneal route. At 3 days post-challenge, serum was collected from all animals to assess the ability of adjuvants to reduce virus titer. Both SLA-Liposomes and QS21-Liposomal adjuvant formulations could protect animals from death (A). Both also reduced D3 serum virus titers (B). However, only SLA-LSQ reduced virus to undetectable levels through D7 (C-D). (*p<0.05, **p<0.005***p<0.0005, ****p<0.0001, One-way ANOVA).
